# Supplementary material for: CombFold: predicting structures of large protein assemblies using a combinatorial assembly algorithm and AlphaFold2
Source: Nat Methods. 2024 Feb 7;21(3):477–87. doi: 10.1038/s41592-024-02174-0 (PMC10927564; doi:10.1038/s41592-024-02174-0)
Supplement: Supplementary file 1 — Supplementary Notes 1 and 2, Fig. 1 and Tables 1–3. [file 41592_2024_2174_MOESM1_ESM.pdf]

nature methods

Article

https://doi.org/10.1038/s41592-024-02174-0

CombFold: predicting structures of large protein assemblies using a combinatorial assembly algorithm and AlphaFold2

In the format provided by the authors and unedited

Supplementary Note 1: Accuracy on Benchmark 4 (CASP15)

We apply CombFold to the seven CASP15 targets containing more than 3,000 amino acids. For proteins with a single chain, the chain was divided into domains according to IUPred3[1], and each domain was used as an input subunit. The automatic pipeline was able to generate Acceptable-quality models for four of the seven targets (Supplementary Fig.1a-d, T1192, H111, T1115, T1165), which translates to a success rate of 57%. For the remaining three targets, using manual division of the chains into subunits as described below, enabled to generate a High-quality model for two additional targets (Supplementary Fig.1f-g, T1169, H1137), increasing the success rate to 86%.

For target H1111, the N-terminal domain of IcrD protein was removed as it was interfering with the rest of the structure. This domain is not present in the published experimental structure. We find that the pairwise interaction generated by AFM and used by our method to compute structures with cyclic symmetry does not always produce ideal cyclic symmetries (Supplementary Fig.1b,c). One future direction is to optimize AFM transformations to ideal symmetries, using symmetric docking tools[2–6] and selecting docked orientations with a pairwise interaction interface most similar to the one produced by AFM.

The remaining three targets did not have sufficient coverage by pairwise AFM interactions to assemble the complexes. For one of the targets (H1114, PDB 7UUS), a partial subcomplex of two HucS and two HucL subunits could be assembled (Supplementary Fig.1e). The other two targets (H1137, T1169) could be predicted with high accuracy by manually dividing their chains into subunits or manually setting subsets of subunits for prediction by AFM (Supplementary Fig.1f,g). Here we describe our prediction of the MceG complex (H1137, PDB 8FEE) for which we submitted to CASP15 a single model which had the highest Contact Agreement Score (QS) of 0.90 among all submissions for this target and a TM-score of 0.87. The pairwise AFM predictions enabled us to identify domains and domain-domain interactions, such as helical domains, roughly the same length in six different chains that are folding into a tube-like structure. Interactions between domains that received a low PAE score (<10.0) were used to generate an interaction graph between the domains, enabling us to group the interacting domains into four groups. The domain fragments we used had overlapping amino acids to enable us to connect consecutive domains by structural alignment. In certain cases, the overlap was of dozens of amino acids, and in other cases, it was of entire domains (Supplementary Table 1). AFM was used to calculate models for each of the four groups, and the overlapping amino acids were used to connect the models of the four groups and obtain the final model (Supplementary Fig.1g).

AFMv2 was able to produce acceptable quality models only for one of these targets. MoLPC was successful only in three targets. CombFold performance is comparable with the top-ranked groups and servers that participated in CASP15 competition (Supplementary Table 3). For almost all targets, different methods were able to produce High-quality models (TM-score > 0.85). There was a notable exception in the case of complex T1115, where CombFold managed to achieve a higher TM-score compared to the top-ranked server's performance (0.72 vs. 0.66). However, human predictors submitted models with higher TM-score.

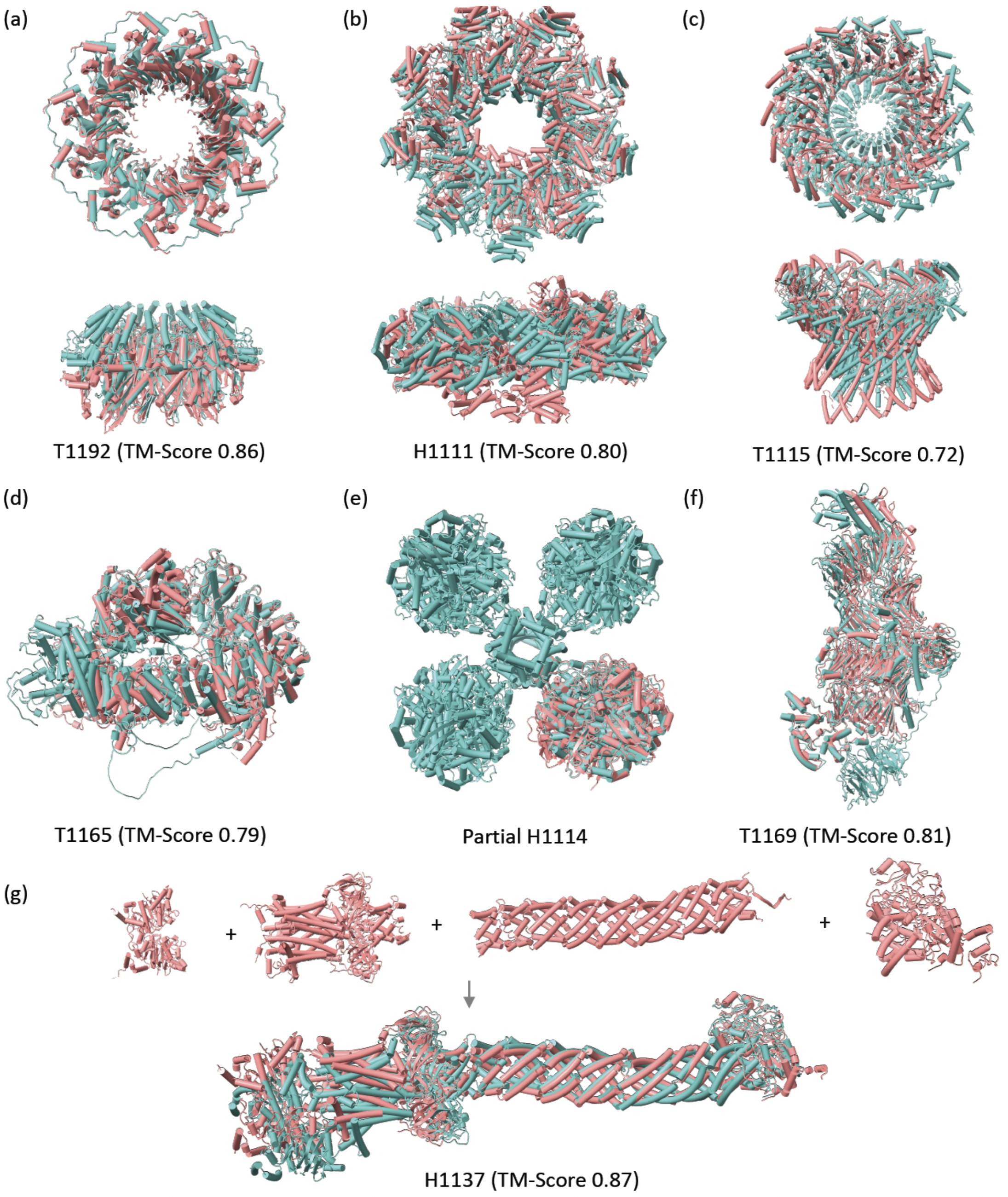

Supplementary Fig. 1. Predicted structure for CASP15 targets. CombFold predictions (coral) are superimposed on the experimental structure (light blue) or top-scoring CASP model if the experimental structure is not released. **(a)** High-quality CombFold model of RAD52 vs. x-ray structure (PDB 5XRZ). **(b)** High-quality CombFold model of YscY-YscX-LcrD vs. x-ray structure (PDB 7QIJ). **(c)** Acceptable-quality CombFold model of stomatin complex vs. top-scoring CASP15 prediction. **(d)** Acceptable-quality CombFold model of Modified ligase Tom1 vs. top-scoring CASP15 prediction. **(e)** High-quality CombFold model of two HucS and two HucL subunits from [NiFe]-hydrogenase Huc complex vs. cryo-EM structure (PDB 7UUS). **(f)** High-quality model of SGS1 vs. cryo-EM structure (PDB 8FJP). **(g)** The four domain groups that were separately modeled by AFM (top) and the final assembly submitted to CASP15 vs. experimental cryo-EM structure (bottom, PDB 8FEE).

Supplementary Note 2: Dataset preparation.

Benchmark 1. To prepare the heteromeric dataset, we downloaded all the PDB entries (up to October 2022 - 195,832 entries) and retained only those that contain at least 5 unique (distinct) chains, resulting in 4,997 entries. Next, we removed entries containing DNA or RNA molecules because they can cause conformational changes upon binding to the protein complex, as well as, entries with short (<50 amino acids) or long chains (>1,500 amino acids), resulting in 1,383 entries. We also filtered entries that contained multiple source organisms, such as host-pathogen interactions, as AFM performance for complexes often depends on the availability of paired alignment, leaving 682 entries. To remove redundancy, the complexes were clustered based on sequence identity. An entry was removed if it had a matching entry with an earlier release date and at least 80% of the chains matching with 70% sequence identity, leaving 213 entries. We also removed all entries released before May 2018 because they were part of the AFM training dataset[7]. The remaining 148 entries were further filtered by resolution (<4 Å). Further, we only kept the entries where the numbering and residue type of ATOM records were consistent with the SEQRES records to enable simple and accurate model validation. This filtering protocol resulted in 35 structures that were used as the test dataset for the pipeline (Benchmark 1, Extended Data Fig. 1a).

Benchmark 2. This benchmark was prepared following a protocol used for Benchmark 1 with several changes. First, the PDB entries used are all entries up to March 2023. Second, instead of filtering entries released before May 2018 (AFMv2 cutoff date), we used September 2021 (AFMv3 cutoff date). Also, we did not filter complexes with chains over 1,500 amino acids. This resulted in 25 complexes (Extended Data Fig. 1b).

Benchmark 3. We relied on a dataset by Bryant et al.[8] that contains 175 complexes. 17 complexes were removed due to incorrectly annotated biological units in the PDB (PDBs 1JYM, 1NLX, 1RVV, 1S3Q, 2E6G, 2EWC, 2VYC, 3HHW, 3KIF, 3R90, 4RSU, 5K2M, 5NFR, 6E7D, 6X04, 6ZEE, 7AJP). In addition, 5 structures of amyloid fibrils were also removed (PDB codes: 6LNI, 6MST, 6OSJ, 6SDZ, 7NRQ) due to the expected compositional and conformational heterogeneity.

Complex Portal. The Complex Portal[9] is a database that curates information from literature on stable, macromolecular complexes. We queried all complexes listed with over 5,000 amino acids with known stoichiometry. Entries with a homologous PDB structure were removed using a stricter threshold for homology to focus on entries that are more likely to be novel. Therefore, we defined a PDB entry as homologous to the Complex Portal entry if at least 66% of the chains were matching with at least 30% sequence identity.

Crosslinks Simulation. We computed a list of all possible K-K crosslinks - that is all pairs of lysine amino acids on different chains with a Cα-Cα distance of less than 25Å, as it is a common distance threshold for crosslinks[10,11]. To select a list of crosslinks for assembly, 10% of those pairs were randomly sampled. To emulate realistic noise, we added 5% false detections of K-K pairs with Cα-Cα distance greater than 40Å.

Supplementary Tables

Chain	Group 1	Group 2	Group 3	Group 4
A		1-175	155-320	300-409
B		1-154	134-302	282-363
C		1-155	135-300	280-384
D		1-167	147-315	295-487
E		1-174	154-318	298-410
F		1-157	137-300	280-423
H+I	All	All		
G+J	All			

Table 1: Subdivision of H1137 into groups of subunits with overlapping domains. Each cell lists the amino acids indexes of the chain present in each model. The division was made so that the helical domain in chains A-F will be calculated as a group (Model 3).

Gene	Change	Structure position
(P) Elp1	Arg696Pro	near interface with Elp6
(L) Elp1	Pro914Leu	interface with Elp2
(P) Elp2	His206Arg	interface with Elp1/Elp3
(P) Elp2	Arg462(Gln/Leu)	core
(P) Elp2	Thr555Pro	core
(L) Elp2	Thr405Ile	core
(P) Elp4	Arg289Trp	interface with Elp6
(L) Elp4	Tyr91Cys	interface with Elp6
(L) Elp4	Leu296Ile	core
(L) Elp6	Leu118Trp	near interface with Elp5

Table 2: ClinVar missense mutations for Elongator holoenzyme complex labeled as pathogenic (P) or likely pathogenic (L).

Target	Comb Fold	AFMv 2	MoLP C	Top-3 human predictors	Top-3 servers

		(Colab Fold)		Zhen g	Venclo vas	Walln er	Yang-multimer	Manifold-E	MULTIC OM_qa
H1111	0.80	0.08	0.52	0.98	0.98	0.09	**0.98**	0.98	0.94
H1114	-	0.12	0.39	0.93	**0.94**	0.13	0.45	0.88	0.24
H1137	0.87	-	0.60	**0.94**	0.82	0.64	0.67	0.88	0.76
T1115	0.72	-	0.46	0.62	**0.90**	-	0.36	0.66	0.38
T1165	0.79	0.75	0.75	-	0.77	0.76	-	**0.80**	0.78
T1169	**0.81**	0.34	0.76	-	0.77	0.50	-	0.74	0.70
T1192	0.86	0.50	0.88	0.88	**0.89**	0.83	0.88	0.88	0.89

Table 3: CASP15 TM-scores for most accurate submitted model for large targets for Top-3 groups and Top-3 servers.

References

1. Erdős, G., Pajkos, M. & Dosztányi, Z. IUPred3: prediction of protein disorder enhanced with unambiguous experimental annotation and visualization of evolutionary conservation. *Nucleic Acids Res.* **49**, W297–W303 (2021).
2. André, I., Bradley, P., Wang, C. & Baker, D. Prediction of the structure of symmetrical protein assemblies. *Proc. Natl. Acad. Sci. U. S. A.* **104**, 17656–17661 (2007).
3. Berchanski, A. & Eisenstein, M. Construction of molecular assemblies via docking: modeling of tetramers with D2 symmetry. *Proteins* **53**, 817–829 (2003).
4. Pierce, B., Tong, W. & Weng, Z. M-ZDOCK: a grid-based approach for Cn symmetric multimer docking. *Bioinformatics* **21**, 1472–1478 (2005).
5. Comeau, S. R. & Camacho, C. J. Predicting oligomeric assemblies: N-mers a primer. *J. Struct. Biol.* **150**, 233–244 (2005).
6. Schneidman-Duhovny, D., Inbar, Y., Nussinov, R. & Wolfson, H. J. Geometry-based flexible and symmetric protein docking. *Proteins* **60**, 224–231 (2005).
7. Evans, R. *et al.* Protein complex prediction with AlphaFold-Multimer. *bioRxiv* 2021.10.04.463034 (2022) doi:10.1101/2021.10.04.463034.
8. Bryant, P. *et al.* Predicting the structure of large protein complexes using AlphaFold and Monte Carlo tree search. *Nat. Commun.* **13**, 6028 (2022).
9. Meldal, B. H. M. *et al.* Complex Portal 2018: extended content and enhanced visualization tools for macromolecular complexes. *Nucleic Acids Res.* **47**, D550–D558 (2019).
10. Rappsilber, J. The beginning of a beautiful friendship: cross-linking/mass spectrometry and modelling of proteins and multi-protein complexes. *J. Struct. Biol.* **173**, 530–540 (2011).
11. Leitner, A. *et al.* Chemical cross-linking/mass spectrometry targeting acidic residues in proteins and protein complexes. *Proc. Natl. Acad. Sci. U. S. A.* **111**, 9455–9460 (2014).
