# Supplementary material for: CombFold: predicting structures of large protein assemblies using a combinatorial assembly algorithm and AlphaFold2
Source: Nat Methods. 2024 Feb 7;21(3):477–87. doi: 10.1038/s41592-024-02174-0 (PMC10927564; doi:10.1038/s41592-024-02174-0)
Supplement: Supplementary file 2 — Reporting Summary [file 41592_2024_2174_MOESM2_ESM.pdf]

nature portfolio

Corresponding author(s): Dina Schneidman
Last updated by author(s): Nov 28, 2023

Reporting Summary

Nature Portfolio wishes to improve the reproducibility of the work that we publish. This form provides structure for consistency and transparency in reporting. For further information on Nature Portfolio policies, see our Editorial Policies and the Editorial Policy Checklist.

Statistics

For all statistical analyses, confirm that the following items are present in the figure legend, table legend, main text, or Methods section.

n/a	Confirmed	
☐	☒	The exact sample size (*n*) for each experimental group/condition, given as a discrete number and unit of measurement
☒	☐	A statement on whether measurements were taken from distinct samples or whether the same sample was measured repeatedly
☒	☐	The statistical test(s) used AND whether they are one- or two-sided<br>*Only common tests should be described solely by name; describe more complex techniques in the Methods section.*
☒	☐	A description of all covariates tested
☒	☐	A description of any assumptions or corrections, such as tests of normality and adjustment for multiple comparisons
☐	☒	A full description of the statistical parameters including central tendency (e.g. means) or other basic estimates (e.g. regression coefficient) AND variation (e.g. standard deviation) or associated estimates of uncertainty (e.g. confidence intervals)
☒	☐	For null hypothesis testing, the test statistic (e.g. *F*, *t*, *r*) with confidence intervals, effect sizes, degrees of freedom and *P* value noted<br>*Give P values as exact values whenever suitable.*
☒	☐	For Bayesian analysis, information on the choice of priors and Markov chain Monte Carlo settings
☒	☐	For hierarchical and complex designs, identification of the appropriate level for tests and full reporting of outcomes
☐	☒	Estimates of effect sizes (e.g. Cohen's *d*, Pearson's *r*), indicating how they were calculated

Our web collection on statistics for biologists contains articles on many of the points above.

Software and code

Policy information about availability of computer code

Data collection	No Software used for data collection.
Data analysis	We have used the following software for data analysis:<br>1. TM-scores were calculated using MMalign (2021-08-16)<br>2. Biopython 1.79 was used for RMSD calculations<br>3. The plots and correlations were calculated using python3 matplotlib 3.53 versions<br>4. The structures were analyzed and visualized using ChimeraX 1.5<br>5. Amber calculations completed using implementation alphafold python package<br><br>CombFold assembly is implemented using C++. The code, Colab notebook, and tutorial for CombFold are available In GitHub:<br>https://github.com/dina-lab3D/CombFold<br><br>Project Code Ocean Capsule:<br>https://codeocean.com/capsule/8791899

For manuscripts utilizing custom algorithms or software that are central to the research but not yet described in published literature, software must be made available to editors and reviewers. We strongly encourage code deposition in a community repository (e.g. GitHub). See the Nature Portfolio guidelines for submitting code & software for further information.

Data

Policy information about availability of data

All manuscripts must include a data availability statement. This statement should provide the following information, where applicable:

- Accession codes, unique identifiers, or web links for publicly available datasets
- A description of any restrictions on data availability
- For clinical datasets or third party data, please ensure that the statement adheres to our policy

In our study we rely on the PDB structures. The PDB codes are available in the github repository.
Scripts and data for manuscript figures are part of the repository. The PDB codes for Benchmarks 1-3 are also part of the repository:
https://github.com/dina-lab3D/CombFold/tree/master/paper_resources/datasets

Human research participants

Policy information about studies involving human research participants and Sex and Gender in Research.

Reporting on sex and gender	N/A
Population characteristics	N/A
Recruitment	N/A
Ethics oversight	N/A

Note that full information on the approval of the study protocol must also be provided in the manuscript.

Field-specific reporting

Please select the one below that is the best fit for your research. If you are not sure, read the appropriate sections before making your selection.

☒ Life sciences ☐ Behavioural & social sciences ☐ Ecological, evolutionary & environmental sciences

For a reference copy of the document with all sections, see nature.com/documents/nr-reporting-summary-flat.pdf

Life sciences study design

All studies must disclose on these points even when the disclosure is negative.

Sample size	We have included in our Benchmarks all the available PDB structures that passed our criteria as detailed in the Data exclusions.
Data exclusions	We have excluded from our Benchmarks all the structures that AlphaFold2 or AlphaFold-multimer was trained on based on the release date and sequence identity cut-offs as described in Methods. This exclusion is required as those structures are expected to be easier to solve and therefore will not be an accurate way to measure our method.
Replication	N/A as there are no experiments in the study and all computations are deterministic and reproducible.
Randomization	N/A as there are no experiments in the study and all computations are deterministic and reproducible.
Blinding	N/A as there are no experiments in the study and all computations are deterministic and reproducible.

Reporting for specific materials, systems and methods

We require information from authors about some types of materials, experimental systems and methods used in many studies. Here, indicate whether each material, system or method listed is relevant to your study. If you are not sure if a list item applies to your research, read the appropriate section before selecting a response.

Materials & experimental systems

n/a	Involved in the study
☒	☐ Antibodies
☒	☐ Eukaryotic cell lines
☒	☐ Palaeontology and archaeology
☒	☐ Animals and other organisms
☒	☐ Clinical data
☒	☐ Dual use research of concern

Methods

n/a	Involved in the study
☒	☐ ChIP-seq
☒	☐ Flow cytometry
☒	☐ MRI-based neuroimaging
